# Novel Concepts of Altered Immunoglobulin G Galactosylation in Autoimmune Diseases

**DOI:** 10.3389/fimmu.2018.00553

**Published:** 2018-03-19

**Authors:** Gillian Dekkers, Theo Rispens, Gestur Vidarsson

**Affiliations:** ^1^Sanquin Research and Landsteiner Laboratory, Department of Experimental Immunohematology, Academic Medical Centre, University of Amsterdam, Amsterdam, Netherlands; ^2^Sanquin Research and Landsteiner Laboratory, Department of Immunopathology, Academic Medical Centre, University of Amsterdam, Amsterdam, Netherlands

**Keywords:** autoimmunity, immune regulation, immunoglobulin G glycosylation, galactosylation, Fc gamma receptor, complement, antibody effector functions

## Abstract

The composition of the conserved N297 glycan in immunoglobulin G (IgG) has been shown to affect antibody effector functions *via* C1q of the complement system and Fc gamma receptors (FcγR) on immune cells. Changes in the general levels of IgG-glycoforms, such as lowered total IgG galactosylation observed in many autoimmune diseases have been associated with elevated disease severity. Agalactosyslated IgG has therefore been regarded and classified by many as pro-inflammatory. However, and somewhat counterintuitively, agalactosylation has been shown by several groups to decrease affinity for FcγRIII and decrease C1q binding and downstream activation, which seems at odds with this proposed pro-inflammatory nature. In this review, we discuss these circumstances where altered IgG galactosylation/glycosylation is found. We propose a novel model based on these observations and current biochemical evidence, where the levels of IgG galactosylation found in the total bulk IgG affect the threshold required to achieve immune activation by autoantibodies through either C1q or FcγR. Although this model needs experimental verification, it is supported by several clinical observations and reconciles apparent discrepancies in the literature, and suggests a general mechanism in IgG-mediated autoimmune diseases.

## Introduction

**Figure 1:**
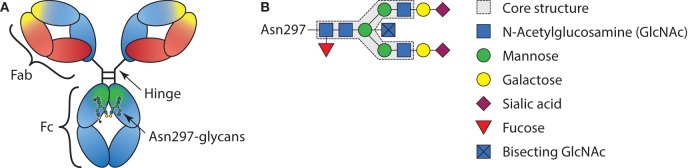
Schematic representation of immunoglobulin G (IgG) structure and glycan composition. **(A)** Schematic representation of antibody with heavy chains and light chains, respectively, in blue and red, with general Y shaped structure. The Fab, Fc, and hinge domains are indicated. Within the Fab domains, the antigen-binding domain is indicated in yellow and within the Fc, the region where the FcγRs and C1q bind is indicated in green. **(B)** Schematic representation of IgG-Fc-N297-glycan with the different sugar groups and their respective positions.

Antibodies are crucial sentinels of the immune system, generated by B cells that sense incoming foreign antigens by their membrane-bound immunoglobulins or B cell receptor (BCR). With each B cell carrying a unique BCR, collectively they are able to respond to virtually any invading substance, let alone a complex pathogen (1). Once recognizing their cognate antigen, each B cell becomes activated and can class switch from the initial IgM and IgD type of BCR, to immunoglobulin G (IgG), IgA, or IgE (2). After maturation to plasmablasts and plasma cells, the B cells start to secrete the acquired BCR in the form of soluble immunoglobulin where it can mount humoral immune responses from complement activation (IgM and IgG) or cellular responses through myeloid and NK cells *via* Fc-receptors (all immunoglobulin types). In plasma, IgG is the most abundant immunoglobulin type found and consists of four subclasses, IgG1, IgG2, IgG3, and IgG4 in order of decreasing abundance. Since the first structures of IgG were solved, it became apparent that these structures are glycoproteins, with a conserved N-linked Fc-glycan attached to the asparagine found at position 297, situated in the constant region of the heavy chain domains (3) (Figure 1). However, it remained enigmatic if, and then how, this rather flexible structure affects the function of IgG. In recent years, these aspects have become ever more clear, although fundamental discrepancies between clinical and experimental observations seem to prevail. These observations and possible resolutions will be discussed further below.

## Mechanism of Glycosylation

**Figure 2:**
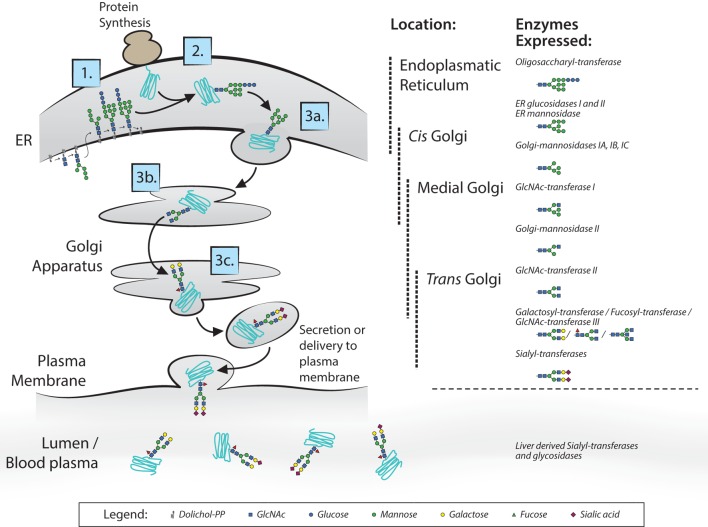
N-glycan biosynthesis. Overview of the N-glycan biosynthesis pathways in the cell and in circulation as also occurs for immunoglobulin G glycosylation (4). In the left panel, the different cell compartments are illustrated with the different steps of synthesis indicated: 1. synthesis of lipid-linked precursor oligosaccharide, 2. *En Bloc* transfer to protein, 3. processing; 3a. initial trimming of sugar residues, 3b. trimming and modification of branch structure, 3c. addition of terminal sugar residues. In the right panel, the different involved glycosidases and glycosyltransferases listed at the position in the endoplasmic reticulum and Golgi apparatus where they proposed to be expressed. Extracellularly, glycosyltransferases and glycosidases are present in circulation, mainly editing the terminal sialic acid groups on glycans.

Most membrane surface proteins and secreted proteins found in plasma are glycosylated. Glycan synthesis starts at the endoplasmic reticulum (ER) when a lipid-linked precursor oligosaccharide is synthesized (Figure 2) (4). In the ER lumen, this precursor is transferred to the Asn site of the protein at accessible residues containing the Asn-X-Ser/Thr motif, where X is any amino acid except proline. Further processing of the glycan then takes place in the ER and Golgi apparatus, which includes trimming and remodeling of the glycan. The cell type-specific spatial and temporal organization of glycosidases and glycosyltransferase expression in ER and Golgi apparatus regulate the final composition of the glycans (4–6). For IgG, assembly of heavy and light chains takes place early in the ER (6). After initial trimming of glucose and mannose groups by the glucosidases and ER mannosidase I in the ER, the whole complex is transported to the *cis*-Golgi (6). The diversity of the glycans derives from several factors; involvement of many different enzymes and substrates in different compartments, variable modification of glycan core structure to bi-, tri-, and tetra-antennary, competition between enzymes for substrates and acceptors, accessibility of the enzymes to the glycan, incomplete processing, and other posttranslational modifications on the same protein (6). For the IgG N-glycans, we know they assemble in a bi-antennary glycan with a core structure of mannose and *N*-acetylglucosamine groups and variable extension of galactose, sialic acid, fucose, and bisecting *N*-acetylglucosamine (bisection) (Figure 1B).

Extracellularly, glycosyltransferases and glycosidases are present in circulation, mainly editing the terminal sialic acid groups on glycans (7). This has been proven to be a functional mechanism in the sialylation of IgG, found in a study where mice with ST6GalT1-deficient B-cells did contain sialylated IgG. This occurs through liver-derived ST6galT1 and platelet-derived CMP-sialic acid as sugar donor, which are present in the circulation (7). Although sialylation of IgG seems to be affected also in plasma, we have observed several immune responses against both red blood cell (RBC) and platelet antigens formed after transfusion or pregnancy, can have markedly different sialylation than total IgG in the same patient (8–11), suggesting that B cells can also have a significant influence on the IgG sialylation in humans. This is supported by mouse work, where they show *in vivo* that overexpression or knockout of sialyltransferase in B-cells attenuates IgG sialylation and disease activity in collagen-induced arthritis (12).

## IgG-Fc Glycosylation in Humans and in Autoimmunity

When analyzing normal IgG repertoire in normal human serum it is found that the overall total glycosylation pattern is, although heterogeneous, generally quite constant, with high fucosylation (96%), low bisection (8%), intermediate galactosylation (40%), and low sialylation (4%) (13). Age and gender are two factors that were found to be correlated with the overall IgG glycosylation patterns. The main variations consist of a decrease in average galactosylation and sialylation and slight increase in bisection associated with higher age (13). The degree of fucosylation is almost 100% shortly after birth (when maternal antibodies have dissipated), after which levels of IgG fucosylation gradually reach ~96% around 20 years of age (14). Infection status, BMI, and epigenetic influences also seem to alter total IgG glycosylation (15–17).

Glycosylation patterns of total IgG have also been observed to temporarily change during certain conditions. During pregnancy, in particular, the degree of galactosylation and sialylation increases, with additional minor decrease in bisection while fucosylation remains stable (18, 19). Furthermore, in autoimmune diseases, changes in total IgG have been detected in, for example, rheumatoid arthritis (RA) (20, 21), inflammatory bowel disease (22), multiple sclerosis (23), myasthenic gravis (24), ankylosing spondylitis, primary Sjögren’s syndrome, psoriatic arthritis (25), and systemic lupus erythematosus (SLE) (26). In all these diseases, a lower degree of total IgG galactosylation is associated with disease progression and flare (20–28). However, the relevance of galactosylation of total IgG, which by definition are not causing the disease, on the disease severity is unknown. In inflammatory autoimmune disorders, such as RA and SLE, IgG autoantibodies presumably play a role in initiating or perpetuating the inflammatory condition (29, 30). In particular in RA, several types of autoantibodies have been identified that target a range of subtle chemical modifications of autologous proteins, including anti-citrullinated protein antibodies (ACPA) and anti-carbamylated protein antibodies—which may be referred to collectively as anti-modified protein response (31). The exact role of the autoantibodies in these diseases has not been fully elucidated as yet, although certain passive transfer mouse models suggest a pathogenic role for ACPAs (32). To our knowledge, the IgG Fc glycosylation patterns have only been determined for disease-associated autoantibodies of a few antigen-specific IgG, including ACPAs and anti-RBC autoantibodies (11, 33–37). In all these diseases, fucosylation is not lowered, even increased for ACPA in RA (11, 33, 34). Several studies suggest similarly low, but variable, levels of galactosylation in the antigen-specific IgG as found in the total IgG (11, 34, 35, 38). However, in two studies, differential changes in glycosylation patterns have been observed between total IgG and specific IgG1 (11, 34), observed in PR3-ACPA and anti-RBC autoantibodies. In both these studies, the galactosylation was variable between the patients, with total IgG galactosylation often diverging from antigen-specific IgG galactosylation. The PR3-ANCA IgG1 antibodies also showed a particularly stable and relatively high galactosylation during relapse, while the total IgG galactosylation was lowered (34). Importantly, this may be a relevant phenomenon that may affect the disease outcome, most likely due to elevated FcγRIIIa and/or FcγRIIIb occupancy which is likely to affect effector functions, as will be discussed in detail in the following sections of this review.

## The Importance of IgG-Fc Glycosylation for FcγR-Mediated Effector Functions

**Figure 3:**
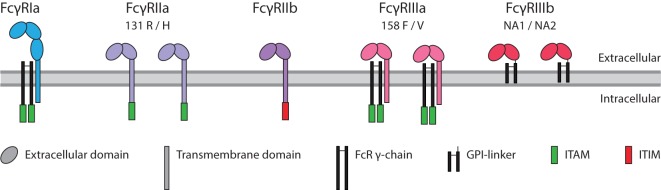
Human FcγR family. The family of human FcγR in with allotypes indicated. Green box: immunoreceptor tyrosine activation motif (ITAM), red box: immunoreceptor tyrosine inhibitory motif (ITIM).

Immunoglobulin G binds with the Fc region to FcγRs. These immune receptors are expressed on myeloid and NK cells of the immune system. Humans express five different FcγR, which can occur in several allotypic variants (39) (Figure 3). Of these five variants, FcγRIa, FcγRIIa, and FcγRIIIa contain an immunoreceptor tyrosine activation motif (ITAM), FcγRIIb an immunoreceptor tyrosine inhibitory motif (ITIM), and FcγRIIIb which is GPI-linked, contains no intracellular signaling domain (40). Binding of IgG to these receptors followed by clustering of the intracellular domains induces an ITAM-mediated signal transduction which can be counteracted by inclusion of the ITIM-containing FcγRIIb, if present (41).

Structurally, the glycan opens up the Fc-portion of IgG and keeps the two CH2 domains at a distance from each other, allowing for interactions with not only FcγR but also C1q that both require similar residues in the IgG for binding (42–44). In addition to the fact that the glycan has been found to show direct, but minor, interactions with the protein backbone of FcγR (43), the IgG Fc-glycan also interacts with a glycan found only in human FcγRIIIa and FcγRIIIb (45, 46) thereby affecting binding affinity of IgG to those receptors. If, and then how, the exact glycan composition—thus not only the mere presence of a glycan—affects binding to the other FcγR or C1q has remained unknown until recently, as discussed further below. The final secreted IgG eventually protects us by opsonizing incoming pathogens. These are then recognized by FcγR, the first component of the complement system (C1q), or both. This can trigger multiple effector functions, such as complement deposition and lysis (47), but also FcγR- and or complement-mediated phagocytosis by myeloid cells (monocytes, macrophages, or neutrophils), trogocytosis (where myeloid cells rupture the membrane of target cells), or FcγR-mediated antibody-dependent cellular cytotoxicity (ADCC) through NK or myeloid cells. These effector functions are currently utilized and have been improved upon by protein and glycan-engineering for many current and future therapeutic antibody approaches. However, these effector functions are also triggered in various allo- and autoimmune diseases (48).

**Figure 4:**
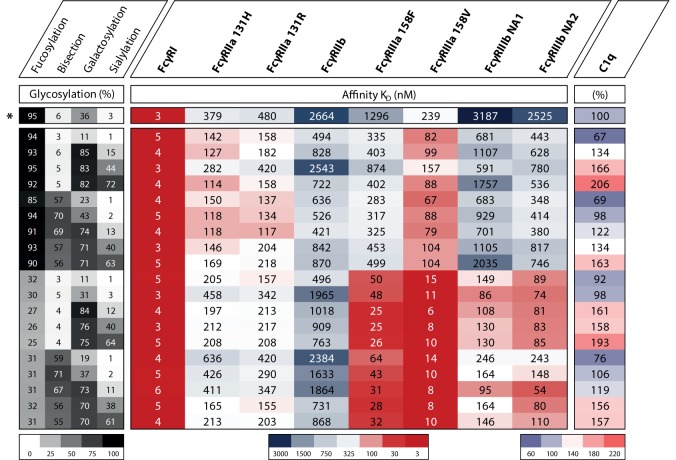
The effect of IgG1 glycovariation and receptor affinity. Heat map displaying various glycoengineered IgG1 and their monomeric affinity in *K*_D_ (nanomolar) for the FcγRs as determined by SPR and relative affinity for C1q as determined by ELISA, data adopted from Dekkers et al. (60). Heat map legends are displayed below. The degree of glycosylation of indicated glycan end groups is colored from white (low) to black (high), the affinity to FcγRs is colored from dark blue (high *K*_D_, low affinity) *via* white to dark red (low *K*_D_, high affinity), the relative affinity for C1q is colored from light blue (low affinity) *via* white to pink (high affinity). *Indicates the normally produced, unmodified IgG1 glycoform which is not glycoengineered.

In humans, we know FcγRIIIa is an important activating FcγR, and of all FcγRs, its affinity for IgG is most influenced by changes in IgG Fc glycosylation. It has been known for over a decade that afucosylation increases affinity of IgG1 to FcγRIIIa and FcγRIIIb (49, 50), and later confirmed for other IgG subclasses (51, 52). Earlier studies have hinted at the possibility that galactosylation may also affect binding to FcγRIIIa in a positive way (53–56), and sialylation in a negative fashion (56–59), but the effect of the other glycans had not been extensively studied. We have recently confirmed that for IgG1 additional galactosylation increases the affinity for FcγRIIIa and FcγRIIIb, but only for afucosylated IgG1 (60), which is found in 6% of IgG1 at the glycopeptide level (13, 61, 62). An overview of the effect of glycovariation on the affinity for FcγRs is displayed in Figure 4. The degree of galactosylation of IgG ranges roughly from 20 to 60% on total IgG and similarly for afucosylated IgG (13, 61, 62). The apparent affinity increases by approximately twofold between afucosylated IgG1 with either 20 vs 70% galactosylation, which corresponds to ~20× or 40× increased affinity compared to fucosylated IgG1, respectively (60).

For bisection and sialylation, we found only small effects on binding to FcγRIII. Additional sialylation of the galactosylated, afucosylated IgG1 caused a slight or no decrease in affinity (56–60). Variation in bisection did not appear to have any effect in the experiments conducted up until now, except for potentially strengthening the negative effect of sialylation on FcγRIIIa and FcγRIIIb-binding (60).

In recent years, it has become apparent that during viral infections of HIV and dengue fever, antigen-specific IgG may contain decreased levels of Fc fucosylation (63, 64). For HIV, this was observed especially for those patients who had a longer disease free survival, the so-called elite controllers, and this correlated with the degree of antibody-mediated cellular viral inhibition (ADCVI) of the patient’s serum. In dengue, a higher degree of afucosylation was more often found in patients with antibody-dependent enhancement of disease (64). For these infections, and possibly more viral infections, the enhanced affinity for FcγRIIIa of the afucosylated antigen-specific IgG indeed enhances ADCC and ADCVI of the virus and virus infected cells (65). For HIV, this rationalizes the better clinical outcome for the patients who have more antigen-specific antibodies with low fucose, but for dengue, the stronger side effects have negative side effects.

Interestingly, the changes in antibody fucosylation and galactosylation of antigen-specific IgGs are also found in alloimmune settings. Examples of this are after blood transfusion, fetal neonatal immune thrombocytopenia (FNAIT) and hemolytic disease of the fetus or newborn (HDFN) (8–10, 66, 67). Both these latter diseases are in a pregnancy setting where the fetus is positive while the mother herself is negative for a paternal antigen on platelets or RBCs, respectively, for FNAIT or HDFN, and thus makes antibodies against the blood cells of the child upon exposure. This can lead to complications and is dangerous for the health of the child and hence it is important to diagnose correctly and timely and also treat accordingly (68).

We have recently shown that glycosylation status of these antibodies matters for pathogenicity (8, 66, 69). The lower degree of fucosylation, in particular, and also increased galactosylation of these antibodies correlate with enhanced disease severity (8–10, 66, 69). The effector mechanism of these antibodies is thought to take place *via* FcγR-bearing cells in the liver and spleen of the fetus, which target the RBC or platelets (70). Previous work has already suggested that FcγRIIIa is the main receptor involved in both RBC and platelet clearance because patients are more likely to carry the high-affinity FcγRIIIa allele (70, 71), now further supported by the observation that IgG with glycosylation patterns that target them to FcγRIIIa with higher affinity also seem to correlate with enhanced disease severity in these diseases (8–10, 66).

As mentioned above, altered glycosylation has been detected and described for many autoimmune diseases (20, 26, 28). This can be either in the total IgG of a patient (i.e., all IgG specificities and not directly related to the disease entity itself) (20–26) or in the IgG specific for the disease (11, 33–37). Most often, a decrease in total IgG Fc-galactosylation has been found and associated with disease progression or severity (5, 20, 22, 26). By contrast, total IgG Fc galactosylation increases during pregnancy, which is clearly associated with disease remission in RA (18, 72–74). It has been shown that FcγRs are important for RA and other autoimmune diseases but axillary involvement of complement is very likely (75, 76). These associations have almost exclusively brought about the hypothesis that agalactosylated IgGs are pro-inflammatory, while highly galactosylated and sialylated IgGs are anti-inflammatory. This anti-inflammatory nature of IgG has been shown in mice not only to stem from binding of hypersialylated IgG to SIGN-R1 (in humans DC-SIGN) (77, 78) but also to the structural homolog and previously identified low affinity IgE-receptor CD23 (77–79). Structural changes in sialylated IgG do not seem to support this model (80–82), and work with sialic-acid enriched IVIg by other groups does not support this notion (83–87). Using detailed glycoengineered IgG (60), we also find no binding of any glycoform to the human receptors (Temming et al. manuscript in preparation). For galactosylation, the exact role of IgG galactosylation and its influence on disease activity also needs to be further elucidated. Importantly, recent affinity data seem at odds with the widespread notion that agalactosylated IgG are pro-inflammatory, given the weaker binding of agalactosylated and afucosylated IgG to FcγRIIIa in comparison to galactosylated, afucosylated IgG as described above. In addition, highly galactosylated IgG has also been associated with enhanced anti-inflammatory properties when present in immune complexes, as it apparently promotes the association of FcγRIIb with Dectin-1 (88).

However, an often overlooked aspect of FcγR binding and subsequent activation through IgG-containing immune complexes or IgG-opsonized cells, is the fact that this occurs in the presence of high concentrations of monomeric (total) IgG in circulation. At these high concentrations (around 10 mg/mL or 60–70 µM, or 7 mg/mL or 40–50 µM of IgG1), exceeding the *K*_D_ for binding of the most common glycoforms of IgG1 to, e.g., FcγRIIa or FcγRIIIa by at least two orders of magnitude (39), most Fc receptors will be bound to monomeric IgG (i.e., a degree of saturation of ca. 98% or more). This is even higher for the high-affinity FcγRIa, which is saturated to an even higher degree, and with less displacement. Upon encounter of, e.g., an FcγR-bearing cell with an opsonized cell providing higher-avidity interactions, the monomeric IgG occupying the FcγR may quickly dissociate and binding of the opsonized cell will take place. How efficient this occurs will depend on the nature of the monomeric IgG (subclass and affinity); which will be strongly influenced by the relative levels of the different Fc glycovariants, but only for FcγRIIIa and FcγRIIIb.

**Figure 5:**
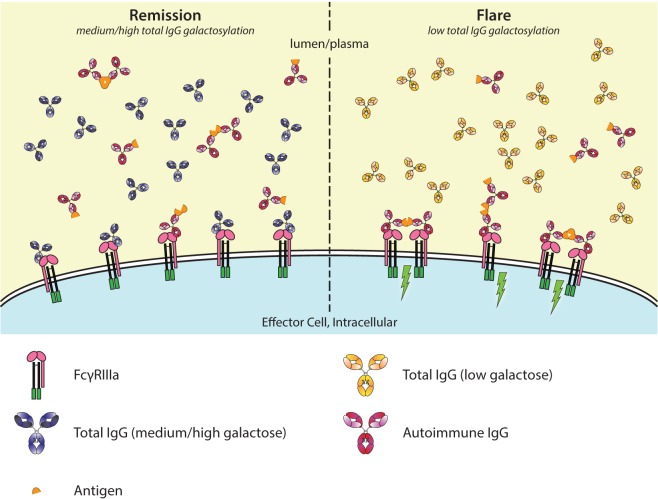
Galactosylation of total immunoglobulin G (IgG) affects IgG-occupation of FcγRIIIa, affecting activation thresholds and flares in autoimmunity. In autoimmune diseases, such as rheumatoid arthritis, disease severity is negatively correlated with the degree of galactosylation. Galactosylation of IgG is important for binding to FcγRIIIa, where—in combination with afucosylation—a higher degree of IgG-Fc galactosylation increases the affinity. Only ~6% of normal serum IgGs is afucosylated. During remission (left), the total IgG galactosylation is relatively high, which prevents autoantibodies to engage the FcγRs. During a flare of disease (right), the total IgG galactosylation is low, which reduces the overall binding affinity of total IgG to FcγRIII, and therefore lowers the threshold for FcγR-activation by allowing more easy access by pathogenic autoantibodies, causing immune activation (green lightning bolt).

The most important glycosylation changes affecting this are again fucosylation and galactosylation. As explained above, we and others have observed that afucosylated, highly galactosylated IgG has a higher affinity for FcγRIIIa and FcγRIIIb. These changes are reflected not only in the activity of NK cell to mediate ADCC *via* FcγRIIIa (53, 55, 60, 89) but also on how irrelevant antibodies, which may bind as monomeric entity to these FcγR, can inhibit the ADCC of specific antibodies (52). This may be relevant, e.g., in the context of RA, where during disease remission, the bulk of antibodies have a normal galactose percentage while during a flare the bulk of antibodies is lowered in galactose, including the afucosylated fraction. Of note, given the ca. 20-fold stronger binding of afucosylated IgG for FcγRIII (49, 60, 90), approximately 50% of IgGs bound to FcγRIII are expected to be afucosylated (given that the concentration of the latter is ca. 20-fold lower). Therefore, comparing a theoretical transition from a flare to remission, an elevated portion of receptor-bound monomeric IgG can be expected to consist of afucosylated, galactosylated IgG with a 2- to 3-fold higher affinity compared to afucosylated, agalactosylated IgG during remission. Under these conditions, immune complexes, in case of autoantibodies almost exclusively fucosylated judging from the current knowledge on ACPA and anti-RBC autoantibodies (11, 34, 38), are expected to have less capacity to displace these higher-affinity IgG from the FcγR. How strong this effect is, will particularly depend on the relative shifts in galactosylation profiles of the total IgG, although galactosylation changes in the autoantibodies themselves may also affect this balance (54–56). Theoretically, immune-complexes therefore have less tendency to cause crosslinking and immune activation if the bulk of IgG show relative elevated IgG-Fc galactosylation, and hence have diminished capacity to cause disease (Figure 5). Overall, the altered glycosylation profiles of autoantibodies may result in altered FcγR-binding and activation, but this may be attenuated by altered glycosylation profiles of non-specific total IgG. We should, of course, realize that all these autoimmune diseases are characterized by multifactorial components, ultimately resulting in disease onset and progression. For RA, this includes the acquirement of multiple disease factors, such as infiltration of immune cells into the joint, ACPA, anti-hinge antibodies, rheumatoid factor (IgM based), increased TNF levels, and complement discussed below (75, 91).

## The Importance of IgG-Fc Glycosylation for Complement-Mediated Functions

Fc glycosylation—the presence of the Fc glycan—is important for classical complement activation, has been known already for a long time (42, 92). In RA, the complement system has been suggested to play an important part of the pathological features, and so have changes in IgG Fc glycosylation are in the galactose end groups (18, 20, 93). The same seems true for many other autoimmune disease where lowered IgG-Fc galactosylation of total IgG correlates with disease severity (5, 11, 20, 22, 26). One study proposed that activation of complement by these agalactosylated IgG species involves specific recognition by the mannose-binding lectin and activation through the lectin pathway of complement activation (94). However, to our knowledge, these results have never been verified. In contrary, we have recently found that IgG agalactosylation—irrespective of all other glycan end groups—does not induce activation of complement *via* the lectin pathway, confirming various other studies on this subject (60, 95, 96). This seems to leave the classical pathway as the main route for enhanced disease activity possibly affected by IgG-Fc glycan variations, by binding of antibody (complexes) to C1q, although deviations from this are likely to exist depending on the disease etiology.

Having established that low galactosylation of total IgG seems to correlate with enhanced disease activity in several autoimmune diseases where complement seem to play an important role (5, 11, 20, 22, 26), has led to the suggestion that agaloctosylated IgG is pro-inflammatory with enhanced complement activity. In contrary, several publications seem now to suggest the opposite to be true. While a few studies found that fucosylation does not affect complement-mediated activity (49, 51, 60), two recent studies suggest that galactosylation of human IgG positively affects C1q binding and downstream activation (60, 97, 98). In our recent study where we screened 20 different and highly defined IgG glycovariants for C1q binding and activation, we found that galactosylation primarily enhanced C1q binding and increased CDC and that additional sialylation increases this effect on the classical complement activation pathway. Bisection showed no affect in any combination with fucose, galactose, or sialylation (60) (Figure 4). Similarly, Quast et al. (97) showed that increased galactosylation enhances C1q binding and CDC in antibody models, where CDC is the main effector function. However, in their model, the addition of sialic acid hampered this enhanced effect (97). The difference in effector function might depend on the nature of the antigen, effector cells, and/or target cells. This is plausible as hexamerization of IgG on the surface of the target is likely needed for proper binding and activation of C1q, and the propensity of different antibodies/antigens to do this may vary as they take on different molecular configurations (99, 100). Much more research on this mechanism is, however, required as we do not know how the specificity of the IgG influences this effect or whether these changes are also relevant in a setting where IgG is already enhanced for CDC by protein engineering (101, 102).

**Figure 6:**
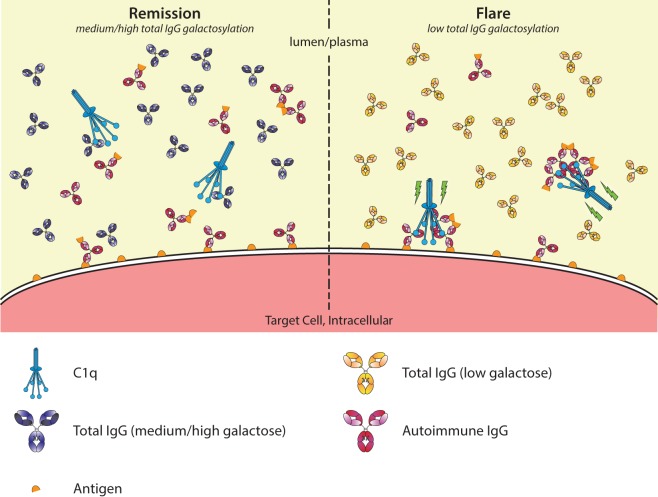
Galactosylation of total immunoglobulin G (IgG) affects C1q occupation and activation in autoimmune remission and flare. In autoimmune diseases, such as rheumatoid arthritis, disease severity is negatively correlated with the degree of galactosylation found in total IgG. Galactosylation of IgG is important for binding to C1q, where a higher degree of IgG Fc galactosylation—and sialylation—increases the affinity. Additionally, C1q requires multimerization/hexamers of IgG for proper activation. During remission (left), C1q-autoantibody engagement might be prevented by preferential interaction with aspecific highly galactosylated IgG, which is abundantly present. During a flare (right), the total IgG galactosylation is low, which reduces the overall threshold of C1q binding, allowing for relatively better binding of pathogenic IgG/immune complexes, allowing activation of the classical complement pathway (green lightning bolt).

It may seem counterintuitive that agalactosylated IgG has been associated with higher pathogenicity while new studies with glycoengineered IgG shows it to have lower potential to activate C1q compared to galactosylated IgG. A possible explanation may be similar to that what we propose for FcγRs (Figure 6). During remission, total IgG galactosylation is relatively high, while during a flare of the autoimmune disease the total IgG galactosylation is relatively low. In the latter case, the threshold for activation of C1q might be lowered due to lower steady-state occupancy by the low-galactosylated IgG. If so, then this could allow for relatively increase in activation of complement by pathogenic IgG complexed by its cognate autoantigen.

Although the binding affinity of monomeric to IgG has been estimated to be very low, i.e., in the 20–100 µM range (103–106), we again have to take into account the exceptionally high concentration of IgG in serum, making this model plausible. Considering IgG1, present at concentrations around 40–50 µM, this translates to ca. 30–70% saturation of C1q. However, care should be taken as measurements of the affinity of monomeric IgG binding to C1q might have been influenced by the presence of trace amounts of aggregates, resulting in an overestimation of the deduced affinities.

Furthermore, we do not know how well these biochemical principles translate into the *in vivo* setting. This could be experimentally determined, but needs high concentrations of both IgG and C1q as the monomeric affinity is generally very low. However, it is important to keep in mind that we do not yet know whether the observed changes in IgG galactosylation in, for example, RA are truly causative of disease flares or a response to the flares. Efforts to investigate the causative association should be undertaken.

## Conclusion

All in all, it was shown that glycan alterations found in IgG seem to be driven by the type of response and have functional consequences. The knowledge that afucosylation imposes better effector functions has already been put to use to enhance the function of therapeutic antibodies used in cancer treatment (107, 108). Additional glycoengineering of the galactose end groups could thus even further improve the functionality of these antibodies. These changes do occur naturally in humans as they are formed in certain immune reactions resulting in stronger humoral immune responses (9, 63, 64, 66, 69). For autoimmune mediated diseases, where changes in galactosylation in both the bulk and the pathogenic antibodies are frequently found, glycosylation changes effects both of binding to FcγR and C1q, which in turn affects their downstream activation. Based on the current evidence, the glycan changes in the bulk of endogenous IgG have the opposite effect on what is observed for antigen-specific IgG. Skewing toward lowered levels of galactose of the bulk IgG lowers its potential to efficiently block both FcγR, but also potentially C1q, giving more room for activation by existing pathogenic IgG-complexes. Eventually, it might be possible to monitor the glycosylation status, especially degree of galactosylation, of the total or disease-specific antibodies in autoimmune diseases. This could help to predict or detect an upcoming flare in autoimmune diseases, enabling intervention early after, or even before symptoms to set in.

## Author Contributions

GD composed and made the figures. GD, TR, and GV wrote and edited the manuscript.

## Conflict of Interest Statement

Sanquin Research conducts Academic research in the field of immno- and hematology. It operates within “Stichting Sanquin Bloedvoorziening,” a not for profit organization that manages the supply-chain of blood and blood products in the Netherlands. The authors declare no competing interests.
